# Towards male sterility in *Cryptomeria japonica* using the male strobilus-specific genes of *C. japonica*

**DOI:** 10.1186/1753-6561-5-S7-P180

**Published:** 2011-09-13

**Authors:** Kurita Manabu, Watanabe Atsushi, Konagaya Ken-ichi, Tsubomura Miyoko, Hirao Tomonori, Ishii Katsuaki, Taniguchi Toru

**Affiliations:** 1 Forest Bio-Research Center, Forestry and Forest Products Research Institute, Japan; 2 Forest Tree Breeding Center, Forestry and Forest Products Research Institute, Japan

## Background

*Cryptomeria japonica* D. Don (sugi) is one of the most important Japanese conifer species. Rapid improvement of the wood trait (e.g., growth speed and wood quality) using a conventional breeding approach is not possible, because breeding of coniferous tree requires a very long time. Genetic modification might be a powerful tool to shorten the time needed to breed trees compared with traditional breeding methods because it is able to induce the favorable traits by introduction of specific genes in trees without unnecessary genetic transitions. But the transfer of foreign genes from GM plants to related plant species by pollen has been cited as an environmental concern. For the purpose of creating male sterile GM *C. japonica*, we attempted to identify genes related to male flower formation.

## Methods

To isolate the male strobilus specific genes, we constructed male strobilus specific SSH libraries based on three different stages according to male strobilus development; early stage, tetrad stage and mature stage. The microarray were designed using 19,259 genes consisting of isolated genes from SSH libraries and ForestGen(FORest EST and GENome database [http://forestgen.ffpri.affrc.go.jp/en/index.html]).We analyzed expression profiling associated with male strobilus development. To isolate the male strobilus specific promoter of male strobilus specific genes, *1009-C47* and *1009-C96*, TAIL-PCR methods were performed. To confirm the tissue specific activity of the promoter regions, the promoter::*GUS* fusions were introduced into *Arabidopsis* and *C. japonica*. Furthermore, we introduced *1009-C47*::*Barnase and 1009-C96::Barnase* to *Arabidopsis* and *C. japonica*. We evaluated the ability of pollen formation of the transgenic *Arabidopsis* that introduced *1009-C47*::*Barnase and 1009-C96::Barnase* construct.

## Results and discussion

The microarray analysis was performed using cDNA of different developmental stages of male strobilus; early stage, microspore mother cell stage, tetrad stage, free spore stage and mature pollen stage. We showed that the strongly expressed genes in each developmental stage were markedly different. The GUS assay revealed that the *1009-C47*::*GUS* showed anther-specific GUS activity in *Arabidopsis* and *C. japonica*. The introduction of *1009-C47*::*Barnase and 1009-C96::Barnase* into *Arabidopsis* led to male sterility phenotype. We showed probability that the *1009-C47* promoter and *1009-C96* promoter are useful for male sterilization of *C. japonica.*

